# The Gambling Establishment and the Exercise of Power: a Commentary on Hancock and Smith

**DOI:** 10.1007/s11469-017-9781-8

**Published:** 2017-06-22

**Authors:** Jim Orford

**Affiliations:** School of Psychology, University of Birmingham, Birmingham, B15 2TT UK

**Keywords:** Gambling harm, Power, Establishment, Discourse

Hancock and Smith’s (2017) paper on the policy influence of the Reno model of gambling problems is very welcome. It contributes significantly to a debate we should be having but which has for a number of years been suppressed under the influence of an apparent gambling policy consensus in which the academic world has been complicit. The use of Advocacy Coalition Theory and the Corporate Political Activity framework in their paper is very helpful. Hancock and Smith describe how the ideology of the Reno model has suited the interests of governments, regulators and the gambling industry, united in a cosy, collaborative relationship whose adherence to the shared model threatens effective responsible provision of gambling. This government/regulator/industry coalition is a powerful one. It is at the centre of what I refer to as the gambling establishment (GE). In the British context, its core consists of a close working alliance of the Government Department for Culture, Media and Sport, the Gambling Commission, the industry-led charity GambleAware and the Responsible Gambling Strategy Board.

One of the most useful ways of understanding how power operates is in terms of power’s various *faces* which differ in the degree to which the exercise of power is hidden (Lukes 2005; Orford 2013). GE exerts its influence most strongly, not through the obvious or *naked* use of power (its first face) but in more subtle ways. The second face of power involves *controlling the agenda*; bringing influence to bear on what topics are discussed and which are not. Hancock and Smith give the Collins et al. (2016), BACTA-funded research as an example of how, assisted by the Reno model, the process of deciding what questions should be asked can function to support the status quo. That was just one example of how British gambling research is being subverted in support of the interests of the powerful GE. The programme of research on EGMs which preceded the Collins et al. study was trailed as research of vital policy importance that would address the controversy surrounding the fixed odds betting terminals (FOBTs). However, in the event, the research focused on problem play and problem players rather than on problem products, not surprisingly since the research was commissioned by the Responsible Gambling Trust (before its name change to GambleAware) and the Chair of the research oversight panel was one of the core Reno model authors. The first reports of the research were presented to an audience largely consisting of industry representatives at a conference held in London in December 2015. In the final panel discussion at the conference, both a senior figure at the Gambling Commission and the Chief Executive of one of the largest bookmaking companies, each stated their conclusion from what they had heard that ‘we should be looking at people not the product’. Since then, some of the most policy-relevant British gambling research has been carried out in-house by the Gambling Commission who have consulted with another of the main Reno authors and by an international financial auditing firm. Not only has the principle of independent research been compromised but also standard procedures for maximising objectivity, such as peer review, are being abandoned in the process.

But power is most effectively exercised, often without debate or even recognition that power is involved at all, if the status quo can be accepted by all concerned, including the academic community. If powerful interests can achieve acceptance of the *discourse* which supports their position, their power is even better secured. This is power’s third face. Hancock and Smith clearly identify a number of key elements: gambling is a legal, regulated form of entertainment or recreation which harms only a small number of players who should be the focus of responsible gambling programmes; the ultimate decision to gamble rests with the individual but to make that decision intelligently, individuals need to be properly informed; the social benefits of gambling exceed the social costs. I have referred elsewhere (Orford 2011) to the GE’s harmless entertainment, ordinary business, freedom to choose, personal responsibility and cultural and economic enhancement discourses. Hancock and Smith add a further element of the Reno model: key responsible gambling stakeholders have similar goals and need to work collaboratively. This is an all-important element of the establishment discourse because it positions the industry as a disinterested partner in the pursuit of public health rather than as a body with a conflict of interest. It follows that the industry is a major stakeholder and rather than be excluded from forums where public health is discussed and debated, it should have a prominent place therein.

Trying to persuade us all to think about the subject in a certain way is tantamount to exerting power over us. This is a form of mental power in which ideas are the currency. Nobel-prize-winning economist and once chief economist at the World Bank, Joseph Stiglitz (2002), is just one of a number of prominent figures who have recognised the crucial importance of the intellectual framework under which policy is made and practised. He writes of the process of capturing the mindset of regulators, something he calls ‘cognitive capture’. As British journalist and broadcaster Owen Jones points out in his book, *The Establishment and How They Get Away with It*, it is about a system of accepted ideas which has such a powerful effect that there is no need to think of it as a planned conspiracy. There is no need to ‘place the blame on “bad” individuals with power. It is the system – the Establishment – that is the problem, not the individuals who comprise it’ (Jones 2015, xvii). In social psychology, system justification theory (SJT), for which there is much evidence, holds that there is ‘a quite basic psychological motivation to legitimise and defend the norms, rules or policies of the social systems which form the status quo within which people live…’ (Orford 2013, p. 95). Because of the expertise afforded to scientists, establishment-supporting academic models, such as the Reno model, constitute a significant part of the system and constitute one of the most important ways in which companies dealing in potentially dangerous products try to co-opt scientists (Babor 2009).

Hancock and Smith imply that responsible gambling (RG) is redeemable but I would disagree. The concept of responsible gambling is a public relations coup for the gambling industry and its supporters. It sounds sufficiently unproblematic to be able to convince most people most of the time, but only just below the surface its message is clear and powerful. Hancock and Smith acknowledge that by ‘downshifting responsibility to individuals’, as they put it, the Reno model obscures the influence of other factors such as deceptive and misleading gambling products. But the Reno model is only part of the system in which the whole idea of RG is the central one, carrying as it does the implications that gambling is essentially unproblematic, that any associated harms are exceptional and can be minimised, and that problems can be localised in a small minority of individuals who can be identified, excluded—preferably self-excluded—and treated.

There are many points at which the GE discourse of RG, and the Reno model which supports it, can be challenged. Indeed, the question has been raised of whether a great deal of modern commercial gambling, though mostly operating within existing laws, is in reality fraudulent because the way it is structured and promoted is designed to give an inflated impression of the chances of winning, emphasising wins and winners, skill and control, disguising losses as wins and advertising misleading ‘return to play’ percentages (Binde 2009; Turner 2011). In the case of gambling, the idea of ‘informed consumers’ is problematic since much of the information—some of it highly technical—about how games operate and what exactly are the odds of winning and losing are not transparent, as Hancock and Smith pointed out, citing the case of misleading Alberta annual reports. To pursue the power analogy, those who engage with continuous, fast and technologically sophisticated modern forms of gambling can be thought of as disempowered. And one of the ubiquitous features, wherever people are disempowered, is the difficulty for the disempowered of grasping structural explanations for their circumstances, the consequent tendency for people to blame themselves and to feel shame and responsibility for the harm they experience (Orford 2008). Those most disempowered by gambling includes family members and others closely affected (Langham et al. 2016).

But there are still wider questions to be asked about the contribution that a much-expanded and government-supported commercial gambling sector might be making to a ‘risk society’ (Beck 1992) generally, or more specifically to an ‘age of chance’ (Reith 1999), or one accepting of their being *winners* and *losers*. What contribution might modern commercial gambling, diverse, easily accessible and vigorously promoted with the encouragement of governments, be making to societies in which inequality and income insecurity have risen, in which austerity is promoted politically and personal and family debt has been encouraged (Değirmencioğlu and Walker 2015)?

Hancock and Smith touch on these bigger issues when they point out that since gambling remains a highly contested moral activity, it may be thought paradoxical to endorse a morally neutral position, as the Reno model does. They wish to challenge the inference that moral convictions have no role in policy debates unless they can be cast in the form of scientific conclusions. This theme is the central one addressed by Skidelsky and Skidelsky (2013) in their book, *How Much Is Enough? Money and the Good Life*, dealing with the role of ethics and values in modern life, in which they argue that the modern version of the liberal state ‘insist[s] on public neutrality between rival conceptions of the good.’ Gambling is a prime case of what they are talking about. Its defence is primarily commercial and any criticism of it must be justified in utilitarian terms. In an ethically neutral world, our views about whether gambling, widely available and promoted, is consonant or not with family, community and national values—the really important kinds of question according to the Skidelskys—is not on the agenda. Meanwhile, there are some obvious beneficiaries of this value neutrality, and powerful interests are promoted. The Reno model is an important part, but only a part, of this subtle exercise of power.
